# Barriers to Clinical Trial Implementation Among Community Care Centers

**DOI:** 10.1001/jamanetworkopen.2024.8739

**Published:** 2024-04-29

**Authors:** Hedyeh Ebrahimi, Sandra Megally, Elana Plotkin, Latha Shivakumar, Nicholas J. Salgia, Zeynep B. Zengin, Luis Meza, Neal Chawla, Daniella V. Castro, Nazli Dizman, Ruma Bhagat, Seila Liv, Xiaochen Li, Adam Rock, Sandy Liu, Abhishek Tripathi, Tanya Dorff, Randall A. Oyer, Leigh Boehmer, Sumanta Pal, Alexander Chehrazi-Raffle

**Affiliations:** 1 City of Hope Comprehensive Cancer Center, Duarte, California; 2 Association of Community Cancer Centers, Rockville, Maryland; 3 Roswell Park Comprehensive Cancer Center, Buffalo, New York; 4 Department of Internal Medicine, Yale University School of Medicine, New Haven, Connecticut; 5 Department of Internal Medicine, MD Anderson Cancer Center, Houston, Texas; 6 Genentech, Inc, South San Francisco, California; 7 City of Hope Orange County Lennar Foundation Cancer Center, Irvine, California; 8 Penn Medicine Ann B. Barshinger Cancer Institute, Lancaster, Pennsylvania

## Abstract

**Importance:**

While an overwhelming majority of patients diagnosed with cancer express willingness to participate in clinical trials, only a fraction will enroll onto a research protocol.

**Objective:**

To identify critical barriers to trial enrollment to translate findings into actionable practice changes that increase cancer clinical trial enrollment.

**Design, Setting, and Participants:**

This survey study included designated site contacts at oncology practices with teams who were highly involved with the Association of Community Cancer Centers (ACCC) Community Oncology Research Institute (ACORI) clinical trials activities, all American Society of Clinical Oncology (ASCO)–ACCC collaboration pilot sites, and/or sites providing care to at least 25% African American and Hispanic residents. To determine participation trends among health care practices in oncology-focused research, identify barriers to clinical trial implementation and operation, and establish unmet needs for cancer clinics interested in trial participation, a 34-question survey was designed. Survey questions were defined within 3 categories: cancer center demographic characteristics, clinical trial characteristics, and referral practices. The survey was distributed through email and was open from June 20 through October 5, 2022.

**Main Outcomes and Measures:**

Participation in and barriers to conducting oncology trials in different community oncology settings.

**Results:**

The survey was distributed to 100 cancer centers, with completion by 58 centers (58%) across 25 states. Fifty-two centers (88%) reported that they conduct therapeutic clinical trials, of which 33 (63%) were from urban settings, 11 (21%) were from suburban settings, and 8 (15%) were from rural settings. Only 25% of rural practices (2 of 8) offered phase 1 trials, compared with 67% of urban practices (22 of 33) (*P* = .01). Respondents noted challenges in conducting research, including patient recruitment (27 respondents [52%]), limited staffing (27 [52%]), and nonrelevant trials for their patient population (25 [48%]). Among sites not offering therapeutic trials, barriers to research conduct included limited infrastructure, funding, and staffing. Most centers (46 of 58 [79%]) referred patients to outside centers for clinical trial enrollment, particularly in the context of late-stage disease and/or disease progression. Only 17 of these sites (37%) had established protocols for patient follow-up subsequent to outside referral.

**Conclusions and Relevance:**

In this national survey study of barriers to clinical trial implementation, most sites offered therapeutic trials, but there were significant disparities in trial availability across care settings. Furthermore, fundamental deficiencies in trial support infrastructure limited research activity, including within programs currently conducting research as well as at sites interested in future clinical research opportunities. These results identify crucial unmet needs for oncology clinics to effectively offer clinical trials to patients seeking care.

## Key Points

**Question:**

What are the most common barriers to clinical trial implementation among community cancer centers, and what strategies might overcome them?

**Findings:**

This survey study assessed 58 cancer centers across 25 states. While most centers (88%) offered therapeutic oncology trials, considerable disparities were observed between different care settings, such as fewer industry-sponsored trials at practices with smaller patient volumes and fewer early-phase trials among rural and suburban practices.

**Meaning:**

These findings indicate that community cancer centers face unique challenges to engaging in clinical trials; support is needed from pharmaceutical companies, health care policymakers, national advocacy groups, and investigators to devise targeted interventions that improve access among these centers and enhance equity across all practice settings.

## Teaser

This survey study investigates critical barriers to trial enrollment to translate findings into actionable practice changes that increase cancer clinical trial enrollment.

## Introduction

Over the past several decades, individuals with cancer have benefited from major breakthroughs in the fields of tumor biology, molecular diagnostics, and antineoplastic therapy. One of the primary drivers of this progress is clinical trials, which at once offer novel interventions while advancing the collective understanding of oncology.^1^

Most patients with cancer receive their care through community settings.^2^ Despite previous studies indicating that more than 70% of patients are willing to participate in clinical trials, fewer than 5% of patients with cancer receive treatment within the context of a clinical trial.^3,4,5^ Previous work has identified several common barriers to clinical trial engagement, such as restrictive eligibility criteria, clinician-related barriers, and negative patient perceptions.^3^

Clinical trial barriers have been shown to vary widely between practice settings. Community practices often face unique challenges relative to academic peers, including limited staffing, lack of clinical trial awareness among both patients and clinicians, and transportation difficulties.^6,7^ Such barriers culminate in low recruitment, which has been implicated as the leading cause of early clinical trial termination.^3,8,9^

Although previous studies have shed light on the barriers faced by community cancer centers in offering clinical trials for their patients, implementation of these findings to improve engagement in cancer research has been sparse and poorly documented. The Association of Community Cancer Centers (ACCC) launched the ACCC Community Oncology Research Institute (ACORI) in 2021 to better understand how cancer programs participate in cancer clinical research, determine common barriers related to clinical trial implementation, and establish the needs of cancer programs that do not currently offer clinical trials so that resources can be developed to improve cancer centers’ participation. The survey study presented herein represents the first phase of ACCC/ACORI’s national clinical trial barriers initiative.

## Methods

### Study Design and Data Collection

A 34-question survey was designed in partnership with ACORI and City of Hope Comprehensive Cancer Center. The survey questionnaire can be found in the eMethods in Supplement 1. Survey design, data collection, and statistical analysis were conducted in accordance with the best practices for survey research from the American Association for Public Opinion Research (AAPOR).^10^ This study was reviewed and determined to meet the criteria for exemption for review and the requirement for informed consent by the institutional review board at City of Hope Comprehensive Cancer Center. Building from our group’s prior experience of evaluating clinical trial barriers specific to Southern California,^11^ the questionnaire herein consisted of three broad categories: cancer center demographic characteristics, clinical trial characteristics, and referral practices. Demographic variables of interest included survey respondents’ role at their center, geographic location, patient volume, academic or nonacademic affiliation, and availability of cancer research at their centers. Respondents were first asked whether their sites conducted cancer clinical research. Those who indicated that they participated in research were then asked about the type of oncology research conducted, annual trial enrollment volume, phase of clinical trials offered, number of industry-sponsored trials, frequency of practices to optimize clinical trial implementation, and challenges in conducting clinical trials. Those who indicated that they did not conduct cancer research were asked about their site’s interest in participating in research and their opinions about barriers to running clinical research at their centers. Next, we focused on patient referrals to outside practices for clinical trials. Respondents who indicated their site referred patients for enrolling in the research were asked about the number of patients referred for participation in clinical trials, factors contributing to their referrals, barriers they faced when making referrals, and their follow-up practice after referring patients to other centers.

The target audience of the survey included priority sites identified by the ACORI task force and project advisory committee. These centers included those determined by the ACCC membership database as having staff who were highly engaged with ACORI clinical trials activities, all American Society of Clinical Oncology (ASCO)–ACCC Collaboration pilot sites, and/or sites providing care to at least 25% African American and Hispanic residents. The survey was distributed to 100 centers via email from ACCC-engaged staff to site-specific contacts. Each respondent was asked to complete the survey on behalf of their respective cancer centers, and a financial incentive of $100 was provided to each respondent for their time. The survey was open from June 20 through October 5, 2022.

### Statistical Analysis

Descriptive statistics were used to report the frequency of responses. Qualitative variables, including cancer center demographics, characteristics of clinical research, challenges experienced in conducting research, and barriers to conducting clinical trials, are provided herein as a number with percentage. The Pearson χ^2^ or Fisher exact tests were used for assessment of associations between the features of practice sites (academic vs nonacademic, rural vs suburban vs urban, and yearly patient volume of ≤1000 vs 1001-5000 vs >5000) and availability of phase 1, 2, and 3 trials as well as the number of available industry-sponsored trials (≤10 vs 11-30 vs >30). Statistical analyses were performed using R version 4.3.0 (R Foundation for Statistical Computing). All reported *P* values are 2-sided and a level of .05 was considered statistically significant.

## Results

**Figure 1.**
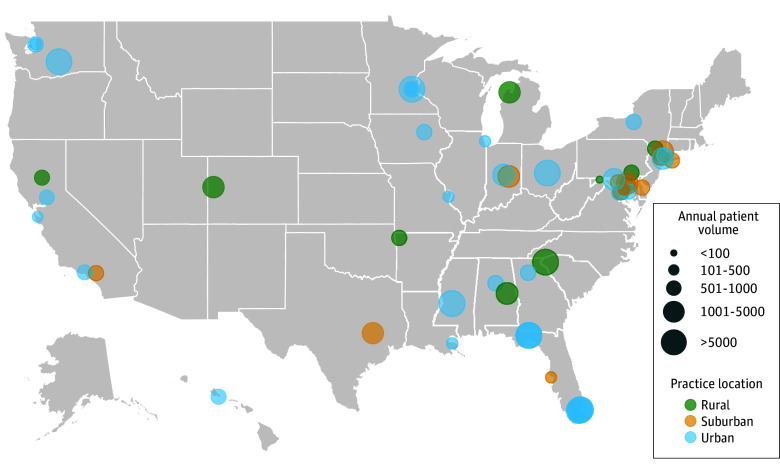
Location of 58 Survey Participants

In this study, the survey was disseminated to 100 cancer centers, of which 58 responded (58%). Participating sites spanned 25 states and the District of Columbia (Figure 1). Respondents and their practice characteristics are summarized in Table 1.

**Table 1.** Characteristics of 58 Survey Respondents and Their Practice Sites

Characteristic	Respondents, No. (%)
Respondents’ primary role	
Advanced practice clinician (NP, CNS, PharmD, PA)	1 (2)
Cancer registrar and accreditation manager	1 (2)
Clinical research associate	3 (5)
Director or administrator	22 (38)
Pharmacist	1 (2)
Physician	9 (16)
Physician investigator	2 (3)
Research coordinator (nonclinician) or data specialist	4 (7)
Research manager or supervisor	13 (22)
Research nurse	2 (3)
Practice setting	
Rural	9 (16)
Suburban	13 (22)
Urban	36 (62)
Geographical location	
Northeast	9 (16)
Midwest	10 (17)
South	29 (50)
West or Pacific	10 (17)
Annual patient volume	
≤1000	23 (40)
1001-5000	26 (45)
>5000	9 (16)
Affiliation with academic research institution	
Academic	32 (55)
Not academic	26 (45)

Abbreviations: CNS, clinical nurse specialist; NP, nurse practitioner; PA, physician assistant.

Of 58 respondents, 52 (90%) indicated that their program currently conducts cancer research. Of these, 31 (60%) were affiliated with an academic center and 21 (40%) had no such affiliation. Most sites were in an urban setting (33 [63%]), followed by suburban (11 [21%]) and rural (8 [15%]). The total annual patient volume was less than 1000 at 18 sites (35%), between 1001 and 5000 at 25 sites (48%), and more than 5000 at 9 sites (17%).

Among the 52 centers that conducted clinical research, 51 sites (98%) offered therapeutic clinical trials, 37 sites (71%) were involved in cancer prevention or screening trials, 35 sites (67%) participated in cancer supportive care trials, and 23 sites (44%) conducted basic science and/or quality improvement research. With respect to annual trial enrollment volume, 18 sites (35%) reported fewer than 100 enrolled patients, 27 sites (52%) reported between 100 and 1000 patients, and 2 sites (4%) reported more than 1000 patients.

Phase 1, 2, and 3 trials were offered in 26 (50%), 46 (88%), and 47 (90%) cancer centers, respectively (Table 2). Stratified by practice setting, the availability of phase 1, 2, and 3 clinical trials was not significantly different between academic and nonacademic sites. Phase 1 trials were available at 48% of academic sites (15 of 31 sites) vs 52% of nonacademic sites (11 of 21 sites) (*P* > .99). Phase 2 trials were available at 81% of academic-affiliated sites (25 sites) vs 100% of nonacademic sites (21 sites) (*P* = .07). Phase 3 studies were available at 94% of academic sites (29 sites) vs 86% of nonacademic sites (18 sites) (*P* = .38). Regarding practice location, phase 1 and 2 trials were offered more frequently in urban settings. Phase 1 trials were available at 25% of rural sites (2 of 8 sites), 18% of suburban sites (2 of 11 sites), and 67% of urban sites (22 of 33) (*P* = .01). Similarly, phase 2 trials were available at 88% of rural (7 sites), 55% of suburban (6 sites), and 100% of urban sites (*P* < .001). Of note, there was no difference observed between practice location and phase 3 trial availability. Phase 1 trials were more commonly offered at sites with an annual practice volume of more than 5000 patients (100% [9 of 9 sites]) than those with fewer than 1000 patients (50% [9 of 18 sites]) or 1001 to 5000 patients (32% [8 of 25 sites]) (*P* = .001). No statistical difference was observed between practice volume size and phase 2 and 3 trial availability.

**Table 2.** Availability of Different Phases of Trials and Industry-Sponsored Trials by Program Type, Location, and Volume Among 52 Practices Conducting Trials

Trial types	Program type, No. (%)		*P* value	Location, No. (%)			*P* value	Annual patient volume, No. (%)			*P* value
	Academic (n = 31)	Nonacademic (n = 21)		Rural (n = 8)	Suburban (n = 11)	Urban (n = 33)		≤1000 (n = 18)	1001-5000 (n = 25)	>5000 (n = 9)	
**Conducting phase 1 trials**											
Yes	15 (48)	11 (52)	>.99	2 (25)	2 (18)	22 (67)	.01	9 (50)	8 (32)	9 (100)	.001
No	16 (52)	10 (48)		6 (75)	9 (82)	11 (33)		9 (50)	17 (68)	0	
**Conducting phase 2 trials**											
Yes	25 (81)	21 (100)	.07	7 (88)	6 (55)	33 (100)	<.001	16 (89)	21 (84)	9 (100)	.64
No	6 (19)	0		1 (13)	5 (46)	0		2 (11)	4 (16)	0	
**Conducting phase 3 trials**											
Yes	29 (94)	18 (86)	.38	8 (100)	9 (82)	30 (91)	.52	14 (78)	24 (96)	9 (100)	.11
No	2 (7)	3 (14)		0	2 (18)	3 (9)		4 (22)	1 (4)	0	
**Available industry-sponsored trials, No.**											
0-10	10 (33)	17 (81)	.002	5 (63)	5 (50)	17 (52)	.67	16 (89)	11 (46)	0	<.001
11-30	6 (20)	2 (9.5)		1 (13)	3 (30)	4 (12.1)		0	7 (29)	1 (11)	
≥31	14 (47)	2 (10)		2 (25)	2 (20)	12 (36)		2 (11)	6 (25)	8 (89)	

Industry-sponsored clinical trials were more prevalent at academic centers than nonacademic centers. At academic sites, 33% offered 0 to 10 industry trials (10 sites), 20% offered 11 to 30 trials (6 sites), and 47% had more than 30 trials (14 sites), whereas 81% of nonacademic sites offered 0 to 10 industry-sponsored trials (17 sites), 10% offered 11 to 30 (2 sites), and 10% offered more than 30 trials (2 sites) (*P* = .002). Additionally, more industry-sponsored trials were available at centers with greater annual patient volumes. At sites with fewer than 1000 patients annually, 89% had fewer than 11 industry-sponsored trials (16 sites) and 11% had more than 30 such trials (2 sites). In contrast, at sites with an annual volume between 1001 and 5000 patients, 46% (11 sites), 29% (7 sites), and 25% (6 sites) had fewer than 11 trials, between 11 and 30 trials, and more than 30 trials, respectively. At centers with more than 5000 patients annually, 11% (1 site) and 89% (8 sites) had between 11 and 30 trials and more than 30 trials, respectively (*P* < .001).

**Figure 2.**
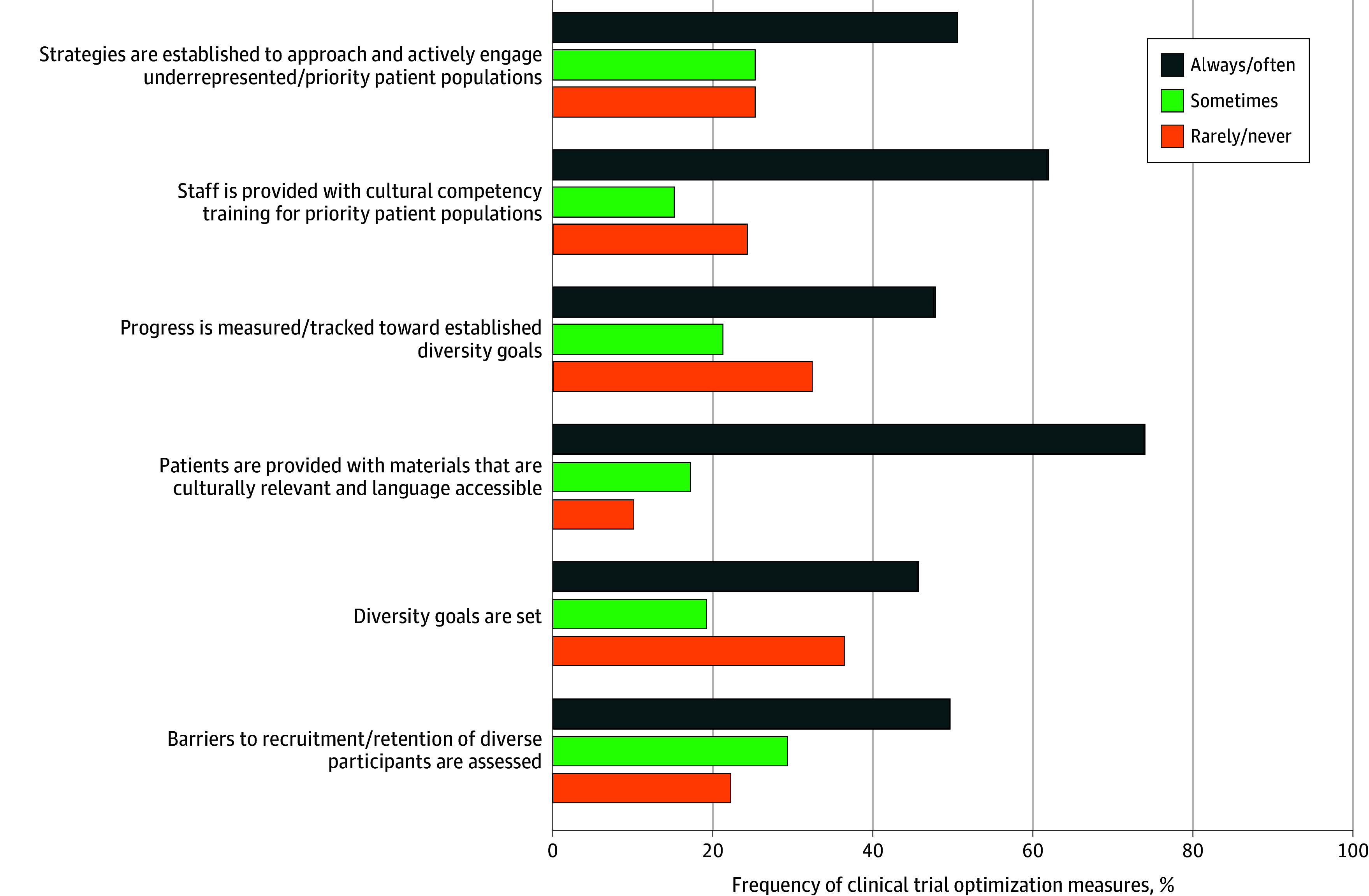
Frequency of Clinical Trial Practices at Cancer Programs Currently Conducting Research A total of 52 site-specific contacts responded to the survey and were conducting clinical trials.

When queried about whether their practice was sufficiently staffed for research activities, 22 respondents (42%) agreed or strongly agreed with the statement. Regarding practices related to clinical trials, the most frequent activities that respondents reported always or often occurring at their programs were (1) providing patients with materials that are culturally relevant and language accessible (38 respondents [73%]), (2) providing staff with cultural competency training for priority patient populations (21 [60%]), and (3) offering logistical supports to patients to enable participation (3 [6%]) (Figure 2).

The most common challenges among programs conducting research were difficulty in recruiting patients (27 [52%]), limited staff for additional research-related activities (27 [52%]), and nonrelevant trials for the patients’ needs (25 [48%]). eTable 1 in Supplement 1 summarizes the reported challenges reported by sites conducting clinical research.

Of the 6 respondents at sites that were not currently conducting research, 4 (67%) mentioned their programs were interested in future clinical research involvement (eTable 2 in Supplement 1). Moreover, they reported limited infrastructure, including information systems, regulatory compliance, institutional review board, data safety monitoring committee, and a clinical trials office, as well as limited funding to hire dedicated research staff and limited staff with the necessary training in research administration as the most common challenges they face.

**Figure 3.**
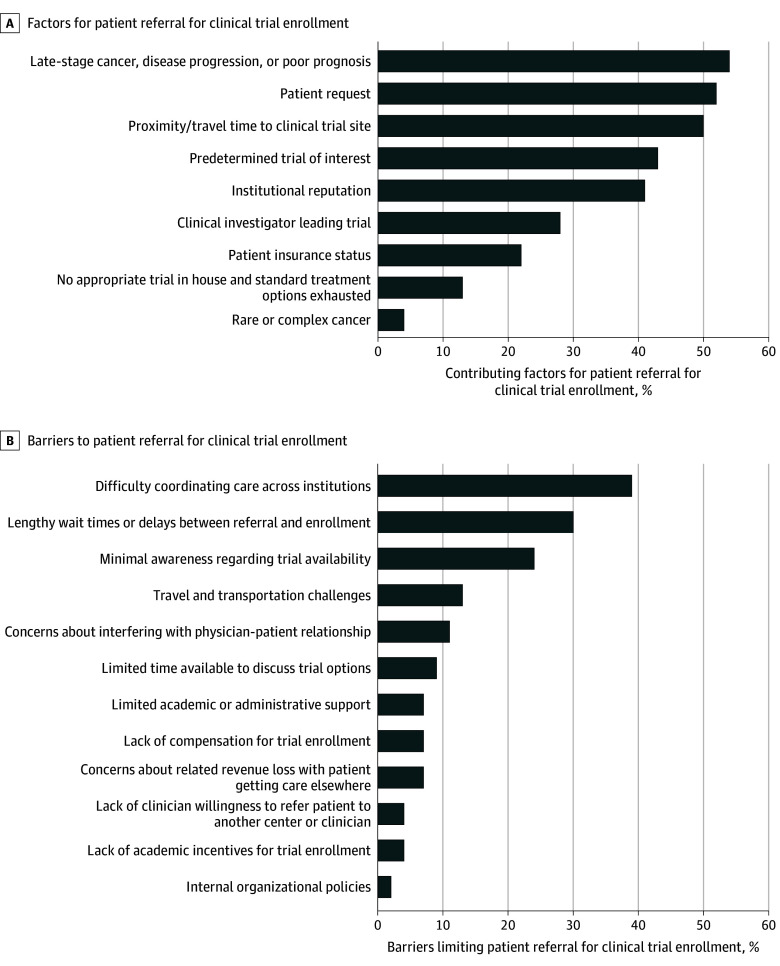
Frequency of Factors Affecting Patients’ Referral to Another Cancer Program for Research Studies

Overall, 46 of the 58 respondents (79%) indicated that their program refers patients to other centers for cancer clinical trials. Of these 46 programs, 12 (26%) referred between 11 and 30 patients, 12 (26%) referred fewer than 11 patients, 2 (4%) referred more than 100 patients, and 20 (44%) were unsure about the number of referred patients at their sites. The most common factors that influenced patient referrals to other cancer centers were late-stage cancer or disease progression (25 [54%]), patient request for referral (24 [52%]), and proximity and travel time to the clinical trial site (23 [50%]) (Figure 3A). In contrast, difficulty with coordinating care across institutions (18 [39%]) and lengthy wait times between referral and enrollment (14 [30%]) were the most common barriers limiting these sites’ referrals (Figure 3B). Twenty-three sites (50%) indicated that they keep patients’ records after referral to another program for clinical trials, and 17 sites (37%) had an established process to follow up with the patient after referral.

## Discussion

This study of cancer treatment centers represents one of the most comprehensive national surveys of clinical trial activity and barriers faced by community cancer centers. Our findings reaffirm numerous long-standing difficulties: the scarcity of available trials, inadequate research-focused staffing, and prolonged intervals between referrals for clinical trials to external institutions. Additionally, we showed that the number of industry-sponsored trials available in community oncology centers and smaller practices were far below the offerings of academic and larger sites. Furthermore, we discerned 2 potential avenues to redress these barriers: increasing early-phase trial availability in rural and suburban areas and enhancing the presence of industry-sponsored trials at practices with smaller annual patient volumes.

Clinical trial barriers have been heuristically grouped into 3 categories: systemic factors, physician-related factors, and patient-related factors.^7^ Systemic obstacles are known to be significant barriers to conducting clinical trials, such as limited information about clinical trial availability and inadequate informational infrastructure.^3^ This study found that limited staffing was the most common barrier faced by community sites not engaged in research, underscoring a need to establish a more robust community-based research infrastructure. Previous studies have delineated potential routes for overcoming systemic barriers to clinical trial conduct, including improving the eligibility criteria for enrollment and refining the reimbursement process for sites conducting trials.^12^ One program that previously assessed systemic problems was the National Cancer Institute Community Cancer Centers Program (NCCCP), a 3-year pilot program launched in 2007 to diminish cancer care disparities at community practices.^13^ Follow-up evaluation of the efficacy of the NCCCP showed improved access to clinical trials and cancer care services as well as improvement in overall community sites’ participation in oncology research.^14,15^ Addressing systemic barriers with initiatives like the NCCCP—and ensuring such efforts are sustainable—will be critical as we collectively chart a path forward.

Clinical research at community cancer centers may also be affected by physician nonparticipation.^16^ Avoidance of discussions about clinical trial enrollment has been attributed to physician preference toward established therapeutic regimens, absence of physician incentives, and lack of time to discuss trial options.^7,17,18,19^ Our study reaffirms that limited physician time is a leading challenge experienced at sites conducting research. The Clinical Trials Navigator developed by the Canadian Cancer Clinical Trials Network was designed to study mitigating such barriers. This pilot program sought to enable health care professionals to search for existing clinical trials more efficiently and ultimately demonstrated a reduction in referral time from nearly 7 days to 1 day.^20^ Implementing navigation platforms demonstrably improves physicians’ ability to discuss existing clinical trials with their patients.

Once a clinical trial is identified for an eligible patient, the patient must then decide whether to participate. Common reasons that patients decline participation in a clinical trial include fear of adverse effects, negative attitude due to hearsay or personal experience, cultural appropriateness, lack of communication between physician and patient, and financial and/or logistical difficulties.^5,7,16,21,22^ We found that patient-initiated requests for trial participation are one of the primary drivers of community sites’ referrals, suggesting that a portion of patients proactively seek inclusion in clinical trials. To address this proactive cohort, the American Cancer Society and Coalition of Cancer Cooperative Groups developed a pilot program entitled Clinical Trials Matching Service.^23^ Between 2007 and 2010, this service enabled patients to speak directly with a clinical trial expert who helped identify a clinical trial best suited to them. This program resulted in an 11% improvement in trial enrollment rate, supporting the utility of patient-driven programs to enhance engagement in clinical trials.

In addition, our survey identified trial barriers that are associated with practice setting, patient volume, and practice location. Phase 1 trials were more common at urban and larger settings, and industry-sponsored trials were more often available at academic and larger practices. Previous studies have also demonstrated that frontline and early-phase trials are less common in community settings.^7,24^ Clinical trial enrollment has also been shown to be significantly lower in rural areas compared with urban settings.^25^ Furthermore, previous research has established that individuals who belong to racial and ethnic minoritized groups are underrepresented in clinical trials. For instance, Unger and colleagues^26^ found that representation of Black patients in industry-sponsored trials was significantly less than in national cooperative group–sponsored trials. These differences in clinical trial access or participation are opportunities to bolster clinical trial engagement as well as enhance the diversity of patient populations in clinical trials. Additionally, our findings reaffirm guidance from the US Food and Drug Administration (FDA) to develop strategies that improve enrollment of participants from underrepresented populations in clinical trials, including but not limited to prespecifying enrollment goals for minority groups and defining metrics that establish whether said participant diversity goals were achieved.

Findings of the current work identified critical points of emphasis for future directives—including those under development by ACCC and the Association of Clinical Research Professionals—to improve clinical trial access in delivery in community oncology practices across the United States. The next phase from this study includes identifying community oncology practices not currently offering clinical trials to provide personalized clinical research conduct training and administrative support to make trials accessible at these sites, particularly in geographic regions and practice settings historically associated with reduced access to clinical research. This is in parallel with the recent FDA guidance to prospectively generate data encompassing more diverse patient populations earlier in the therapeutics development program. Taken together, our comprehensive interrogation of clinical trials in community oncology practices reaffirms existing barriers to research activity, identifies novel barriers to trial conduct and patient enrollment, and establishes a roadmap for ongoing efforts to address these historical barriers to clinical trials in nonacademic settings.

### Limitations

Our study has several limitations. Although our survey response rate was higher than is typical of previous studies, we nonetheless were unable to gather information from all 50 states, which thereby reduces our generalizability. A dedicated investigation designed to assess the root causes of nonresponsive sites to the invitation to participate in the present survey can elucidate another significant set of obstacles pertaining to involvement in any type of research study. Of the responses we did receive, there was a skew toward urban practices compared with suburban and rural practices, hampering comparisons between these different demographic groups. Continued efforts of multiple organizations, including the ACCC/ACORI and the ASCO, seek to directly address these limitations and further enhance clinical trial access through implementation of training programs to enhance trial recruitment at community sites and improve diversity and inclusion efforts in trial enrollment.^27,28,29^ Additionally, we had only a small proportion of practices report that they did not conduct research, which limits our ability to derive robust conclusions about this subgroup. Further studies are needed to specifically focus on practice sites that have never conducted any form of research studies. Moreover, as previously highlighted, there is an ongoing initiative at ACCC dedicated to prioritizing the provision of essential support to community practice sites lacking or having a limited number of clinical trials within their facilities.

## Conclusions

Our nationwide survey study of clinical trial access reaffirmed certain previously identified barriers, including difficulty finding existing clinical trials, understaffing for research purposes, and lengthy wait times after referrals to outside institutions. We also identified several opportunities for potential improvements, such as increasing early-phase clinical trials in rural and suburban areas and increasing access to industry-sponsored trials at practices with smaller annual patient volumes. Our findings informed a comprehensive platform to be developed by ACCC/ACORI that aims to improve community cancer center participation in clinical research for the benefit of patients with cancer from underrepresented geographic, socioeconomic, racial, and ethnic groups. This encompasses the ongoing second phase of the initiative within ACCC/ACORI, aiming to provide exclusive assistance and guidance to community practices with restricted availability of trials for their patients.

## Notes

Supplement 1. **eTable 1.** Clinical Research Challenges Experienced in 52 Sites Currently Conducting Research **eTable 2.** Barriers to Conducting Clinical Trials in 6 Sites Not Currently Involved in Research **eMethods.** Survey Questionnaire

Supplement 2.
Data Sharing Statement
