# Supplementary material for: Barriers to Clinical Trial Implementation Among Community Care Centers
Source: JAMA Netw Open. 2024 Apr 29;7(4):e248739. doi: 10.1001/jamanetworkopen.2024.8739 (PMC11059033; doi:10.1001/jamanetworkopen.2024.8739)
Supplement: Supplement 1. — eTable 1. Clinical Research Challenges Experienced in 52 Sites Currently Conducting Research eTable 2. Barriers to Conducting Clinical Trials in 6 Sites Not Currently Involved in Research eMethods. Survey Questionnaire [file jamanetwopen-e248739-s001.pdf]

Supplemental Online Content

Ebrahimi H, Megally S, Plotkin E, et al. Barriers to clinical trial implementation among community care centers. *JAMA Netw Open.* 2024;7(4):e248739. doi:10.1001/jamanetworkopen.2024.8739

eTable 1. Clinical Research Challenges Experienced in 52 Sites Currently Conducting Research

eTable 2. Barriers to Conducting Clinical Trials in 6 Sites Not Currently Involved in Research

eMethods. Survey Questionnaire

This supplemental material has been provided by the authors to give readers additional information about their work.

© 2024 Ebrahimi H et al. *JAMA Network Open.*

eTable 1. Clinical Research Challenges Experienced in 52 Sites Currently Conducting Research

Challenges	Number (%)
Available trials do not match patient population	25 (48%)
Competing cancer programs in local area	12 (23%)
Difficulty recruiting patients	27 (52%)
Difficulty retaining patients enrolled in trials	6 (12%)
Difficulty working with payers to cover clinical trial costs	14 (27%)
Limited access to information about clinical trials that could be opened at site	7 (13%)
Limited clinician interest	15 (29%)
Limited clinician time	20 (38%)
Limited familiarity with components of trial implementation	2 (4%)
Limited operating finances to open or run trials	9 (17%)
Limited infrastructure	10 (19%)
Limited number of existing staff to feasibly absorb additional research-related activities	27 (52%)
Limited staff with necessary training in research administration and conduct	14 (27%)
Limited or strained relationship between organization and broader community	1 (2%)
Other	8 (15%)

© 2024 Ebrahimi H et al. *JAMA Network Open.*

eTable 2. Barriers to Conducting Clinical Trials in 6 Sites Not Currently Involved in Research

Barriers	Number (%)
**Organizational-level barriers**	
Competing cancer practices or programs in local area	0 (0%)
Lack of collective confidence to implement a research program	1 (17%)
Lack of organizational support for grant proposals and/or managing grant funds	2 (33%)
Lack of relationships for networking/referrals	1 (17%)
Lack of information about available research opportunities	3 (50%)
Lack of support from administrators/management for establishing a research program	3 (50%)
Limited infrastructure (e.g., information systems, regulatory compliance, IRB, data safety monitoring committee, clinical trials office)	4 (67%)
Limited incentives from trial sponsors to participate	1 (17%)
Limited operating finances to open or run trials	3 (50%)
Organizational structure and policies	2 (33%)
**Care team-level barriers**	
Individual multidisciplinary team members' beliefs about research and fit with values/mission of organization	0 (0%)
Lack of clinical investigator experience securing research grants	3 (50%)
Limited access to information about clinical trials which could be opened at site	1 (17%)
Limited clinician interest	0 (0%)
Limited clinician time	1 (17%)
Limited familiarity with components of trial implementation (e.g., IRB compliance, regulatory requirements, data safety monitoring)	2 (33%)
Limited funding to hire dedicated research staff	4 (67%)
Limited number of existing staff to feasibly absorb additional research-related activities	3 (50%)
Limited staff with necessary training in research administration and conduct	4 (67%)
Lack of diverse staff that is representative of priority populations	1 (17%)
Lack of provider time to discuss clinical trials with patients and/or identify trials	0 (0%)
**Patient-level barriers**	
Available trials do not match patient population	2 (33%)
Difficulty recruiting patients	1 (17%)
Difficulty retaining patients enrolled in trials	2 (33%)
Difficulty working with payers to cover clinical trial costs	2 (33%)
Lack of culture or expectation around research acceptance and engagement	1 (17%)
Lack of logistical support available to enable research participation (e.g., travel funds for extra visits, trial-related out-of-pocket costs)	2 (33%)
Limited or strained relationship between organization and broader community	1 (17%)
Not sure	1 (17%)

© 2024 Ebrahimi H et al. *JAMA Network Open.*

eMethods. Survey Questionnaire

Q1 Please indicate your cancer program/practice state.

Q2 Which of the following best describes your primary role? (Select one.)

- ○ Physician
- ○ Advanced Practice Provider (NP, CNS, PharmD, PA)
- ○ Physician investigator
- ○ Oncology Fellow
- ○ Director/Administrator
- ○ Research Nurse
- ○ Nurse (non-research)
- ○ Navigator (nurse or other discipline)
- ○ Research Manager/Supervisor
- ○ Research Coordinator (non-clinician)
- ○ Clinical Research Associate

Other (please specify):

Q3 Which of the following best describes the setting of your cancer program? (Select one.)

- ○ Rural
- ○ Suburban (the outlying district of a city)
- ○ Urban (a town or a city)

© 2024 Ebrahimi H et al. *JAMA Network Open.*

Q4 Approximately how many new patients with cancer does your program receive annually?

- ○ Less than 100
- ○ Between 101-500
- ○ Between 501-1000
- ○ Between 1001-5000
- ○ More than 5000

Q5 Which of the following patient demographic information does your cancer program systematically obtain and document? (Select all that apply.)

- ☐ Race
- ☐ Ethnicity
- ☐ Sexual Orientation
- ☐ Gender Identity
- ☐ Other (please specify):
- ☐ None of the above
- ☐ Not sure

Q6 Which of the following categories describe your cancer program/practice? (Select all that apply.)

- ☐ Community Cancer Program (CCP)
- ☐ Comprehensive Community Cancer Program (CCCP)
- ☐ NCI-Designated Cancer Center
- ☐ NCI-Designated Comprehensive Cancer Center
- ☐ NCI Community Oncology Research Program (NCORP) Site
- ☐ Minority-Underserved NCORP Site
- ☐ Private/Physician Practice
- ☐ Veterans Affairs Cancer Program (VACP)

© 2024 Ebrahimi H et al. *JAMA Network Open.*

☐ Other (specify): ____

Q7 Is your cancer program affiliated with an academic research institution?

○ Yes (which one?): ____

○ No

○ N/A- we are an academic cancer program

Q8 Does your cancer program have a Patient and Family Advisory Council (PFAC) or similar?

○ Yes

○ No

○ Not sure

Q9 Does your cancer program currently conduct cancer clinical research?

○ Yes

○ No

○ Not sure

Q10 Which types of cancer clinical research are conducted at your cancer program? (Select all that apply.)

☐ Cancer clinical treatment trials (i.e., trials involving anti-cancer treatments)

☐ Cancer supportive care trials

☐ Cancer prevention and/or screening trials

☐ Basic science cancer research

☐ Psychosocial and/or behavioral research

☐ Implementation research (i.e., studying methods that support the application of research findings into practice)

☐ Quality improvement research

☐ Other (please specify):

© 2024 Ebrahimi H et al. *JAMA Network Open.*

☐ None of the above

☐ Not sure

Q11 Is your cancer program part of or affiliated with any of the following research networks? (Select all that apply.)

☐ Academic Research Network (please specify): ____

☐ NCI Clinical Trials Network Group (NCTN) – Lead Academic Participating Site (LAPS)

☐ NCI Clinical Trials Network Group (NCTN) – National Community Oncology Research Program (NCORP) Site

☐ NCI Clinical Trials Network Group (NCTN) – Other

☐ OneOncology Research Network (OneR)

☐ Sarah Cannon

☐ US Oncology

☐ Other (please specify):

☐ We are not part of a research network

☐ Not sure

© 2024 Ebrahimi H et al. *JAMA Network Open.*

Q12 Please indicate your level of agreement with the following statement:
Currently, our cancer program has sufficient staffing to support all research related activities across our program.

- Strongly disagree
- Disagree
- Neither disagree or agree
- Agree
- Strongly agree

Q13 On average, how many participants does your cancer program enroll each year on cancer trials? (Consider the annual average enrolled onto trials over the previous 3 years or indicate "not sure.")

Q14 Which phases of trials are offered by your cancer program? (Select all that apply.)

- Phase 1
- Phase 2
- Phase 3
- Phase 4
- None of the above
- Not sure

Q15 Approximately how many industry-sponsored cancer clinical treatment trials (Phases 1-4) does your cancer program currently have open for accrual?

- N/A (site does not participate in industry-sponsored trials)
- 0 (no industry-sponsored trials are currently open for accrual)
- 1 to 10
- 11 to 20
- 21 to 30
- 31 or more
- Not sure

© 2024 Ebrahimi H et al. *JAMA Network Open.*

Q16 Please indicate the frequency with which the practices below occur at your cancer program as they relate to clinical trials.

	Never	Rarely	Sometimes	Often	Always	Not Sure
**Diversity goals are set (e.g., study-specific and priority patient populations)**	○	○	○	○	○	○
Progress is measured/tracked toward established diversity goals	○	○	○	○	○	○
**Barriers to recruitment/retention of diverse participants are assessed**	○	○	○	○	○	○
Strategies are established to approach and actively engage underrepresented/priority patient populations	○	○	○	○	○	○
**Staff is provided with cultural competency training for priority patient populations**	○	○	○	○	○	○
Patients are provided with materials that are culturally relevant and language accessible (e.g., informed consent form, recruitment materials, outcomes assessments)	○	○	○	○	○	○
**Logistical supports are offered to patients to enable participation (e.g., transportation services, lodging, childcare during appointments, stipends)**	○	○	○	○	○	○
Appointments are offered outside of normal business hours (i.e., early arrivals, after-hours and/or weekends)	○	○	○	○	○	○

Q17 What challenges are experienced by your cancer program related to conducting cancer clinical research? (Select all that apply.)

© 2024 Ebrahimi H et al. *JAMA Network Open.*

- ☐ Available trials do not match patient population
- ☐ Competing cancer programs in local area
- ☐ Difficulty recruiting patients (e.g., ask but patients refuse)
- ☐ Difficulty retaining patients enrolled in trials
- ☐ Difficulty working with payers to cover clinical trial costs
- ☐ Limited access to information about clinical trials that could be opened at site
- ☐ Limited clinician interest
- ☐ Limited clinician time
- ☐ Limited familiarity with components of trial implementation (e.g., IRB compliance, regulatory requirements, data safety monitoring)
- ☐ Limited operating finances to open or run trials
- ☐ Limited infrastructure (e.g., information systems, regulatory compliance, IRB, data safety monitoring committee, clinical trials office)
- ☐ Limited number of existing staff to feasibly absorb additional research-related activities
- ☐ Limited staff with necessary training in research administration and conduct
- ☐ Limited or strained relationship between organization and broader community
- ☐ Other (please specify):
- ☐ Not sure

Q18 Is your cancer program interested in conducting cancer clinical research at your practice?

- ○ Yes
- ○ No
- ○ Not sure

Q19 What **organizational-level barriers** to conducting clinical treatment trials are experienced at your practice/program? (Select all that apply.)

© 2024 Ebrahimi H et al. *JAMA Network Open.*

☐ Competing cancer practices or programs in local area

☐ Lack of collective confidence to implement a research program

☐ Lack of organizational support for grant proposals and/or managing grant funds

☐ Lack of relationships for networking/referrals

☐ Lack of information about available research opportunities

☐ Lack of support from administrators/management for establishing a research program

☐ Limited infrastructure (e.g., information systems, regulatory compliance, IRB, data safety monitoring committee, clinical trials office)

☐ Limited incentives from trial sponsors to participate

☐ Limited operating finances to open or run trials

☐ Organizational structure and policies

☐ Other (please specify):

☐ Not sure

Q20 What **care team-level barriers** to conducting clinical treatment trials are experienced at your practice/program? (Select all that apply.)

☐ Individual multidisciplinary team members' beliefs about research and fit with values/mission of organization

☐ Lack of clinical investigator experience securing research grants

☐ Limited access to information about clinical trials which could be opened at site

☐ Limited clinician interest

☐ Limited clinician time

☐ Limited familiarity with components of trial implementation (e.g., IRB compliance, regulatory requirements, data safety monitoring)

☐ Limited funding to hire dedicated research staff

- [ ] Limited number of existing staff to feasibly absorb additional research-related activities
- [ ] Limited staff with necessary training in research administration and conduct
- [ ] Lack of diverse staff that is representative of priority populations
- [ ] Lack of provider time to discuss clinical trials with patients and/or identify trials
- [ ] Other (please specify):
- [ ] Not sure

Q21 What **patient-level barriers** to conducting clinical treatment trials are experienced at your practice/program? (Select all that apply.)

- [ ] Available trials do not match patient population
- [ ] Difficulty recruiting patients
- [ ] Difficulty retaining patients enrolled in trials
- [ ] Difficulty working with payers to cover clinical trial costs
- [ ] Lack of culture or expectation around research acceptance and engagement
- [ ] Lack of logistical support available to enable research participation (e.g., travel funds for extra visits, trial-related out-of-pocket costs)
- [ ] Limited or strained relationship between organization and broader community
- [ ] Other (please specify):
- [ ] Not sure

Q22 Does your cancer program refer patients to other centers for cancer clinical trials?

○ Yes

○ No

○ Not sure

© 2024 Ebrahimi H et al. *JAMA Network Open.*

Q23 At your cancer program, which **factors influence** patient clinical trial referral to another cancer program/center? (Select all that apply.)

- ☐ Institutional reputation
- ☐ Clinical investigator leading trial
- ☐ Late stage cancer, disease progression, or poor prognosis
- ☐ Predetermined trial of interest
- ☐ Proximity/travel time to clinical trial site
- ☐ Patient insurance status
- ☐ Patient request
- ☐ Other (please specify):
- ☐ None of these
- ☐ Not sure

Q24 Which of the following **barriers limit** the ability to refer patients to clinical trials at another cancer program/center? (Select all that apply.)

- ☐ Concerns about interfering with physician-patient relationship
- ☐ Concerns about related revenue loss with patient getting care elsewhere
- ☐ Difficulty coordinating care across institutions
- ☐ Internal organizational policies
- ☐ Lack of academic incentives for trial enrollment
- ☐ Lack of compensation for trial enrollment
- ☐ Lack of clinician willingness to refer patient to another center or provider
- ☐ Lengthy wait times for providers
- ☐ Limited academic or administrative support

© 2024 Ebrahimi H et al. *JAMA Network Open.*

- [ ] Limited time available to discuss trial options
- [ ] Minimal awareness regarding trial availability
- [ ] Other (please specify):
- [ ] None of the above
- [ ] Not sure

Q25 Does your cancer program maintain a record of patients who are referred to other cancer programs/centers for clinical trials?

- ○ Yes
- ○ No
- ○ Not sure

Q26 Please indicate how patient referrals for clinical trials are documented at your cancer program. (Select all that apply.)

- [ ] Free text in patient chart
- [ ] Discrete electronic health record (EHR) data field
- [ ] Other (please specify):
- [ ] Not sure

Q27 Approximately how many patients does your cancer program refer per year to other cancer programs/centers for clinical trials? (Please provide your best estimate, or indicate "not sure.")

Q28 Does your site have a process in place for following up with patients after they have been referred to other cancer programs/centers for clinical trials?

- ○ Yes
- ○ No
- ○ Not sure

© 2024 Ebrahimi H et al. *JAMA Network Open.*

Q29 How often does your site follow up with providers at other cancer programs/centers about patients you refer to them for clinical trials?

- ○ Never
- ○ Rarely
- ○ Sometimes
- ○ Often
- ○ Always
- ○ Not sure

Q30 How often do providers at other cancer programs/centers follow up with referring providers at your site about patients that have been referred for clinical trials?

- ○ Never
- ○ Rarely
- ○ Sometimes
- ○ Often
- ○ Always
- ○ Not sure

Q31 How informed do you feel about clinical trials that are currently being offered at cancer programs/centers in your region?

- ○ Not at all informed
- ○ Somewhat informed
- ○ Very well informed
- ○ N/A – there are no other cancer programs in my region and/or we do not need to refer patients

Q32 What types of support could ACCC offer that would be most helpful to further support capacity to conduct clinical research at your cancer program? (Select all that apply.)

- ☐ Communication about clinical research opportunities
- ☐ Connection to clinical trial sponsors
- ☐ One-on-one consultation/mentorship from another cancer program that conducts research
- ☐ Community of practice or similar model

© 2024 Ebrahimi H et al. *JAMA Network Open.*

- [ ] Grant writing skills training to support/build research program
- [ ] Other, please share additional ideas:
- [ ] No support needed

Q33 Are you willing to allow ACCC to share your name and email address with trial sponsors so that they may reach out to you about opportunities?

- [ ] Yes
- [ ] No
- [ ] Additional Comments/Thoughts/Questions:

Q34 Upon completion of this survey, you will be eligible for an incentive. Would you like to receive it?

- ○ Yes, send to the e-mail I provided
- ○ Yes, send to an alternate e-mail
- ○ No

© 2024 Ebrahimi H et al. *JAMA Network Open.*
