# Supplementary material for: Barriers to Clinical Trial Implementation Among Community Care Centers
Source: JAMA Netw Open. 2024 Apr 29;7(4):e248739. doi: 10.1001/jamanetworkopen.2024.8739 (PMC11059033; doi:10.1001/jamanetworkopen.2024.8739)
Supplement: Supplement 2. — Data Sharing Statement [file jamanetwopen-e248739-s002.pdf]

Data Sharing Statement

Ebrahimi. Barriers to Clinical Trial Implementation Among Community Care Centers. *JAMA Netw Open*. Published April 29, 2024. doi:10.1001/jamanetworkopen.2024.8739

Data
Data available: Yes
Data types: Deidentified participant data
How to access data: Please email achehraziraffle@coh.org to request access to deidentified participant data.
When available: With publication

Supporting Documents
Document types: None

Additional Information
Who can access the data: Anyone requesting the data
Types of analyses: For any purpose
Mechanisms of data availability: With a signed data access agreement
